# Supplementary material for: Communication patterns in decision-making consultations between patients with advanced cancer and medical oncologists: A qualitative observational study
Source: PLoS One. 2026 Apr 7;21(4):e0346036. doi: 10.1371/journal.pone.0346036 (PMC13056162; doi:10.1371/journal.pone.0346036)
Supplement: S1 Table — (DOCX) [file pone.0346036.s001.docx]

**Supplementary 1 Study team and reflexivity**

| **Study team members’ initials** | **Education/Profession** | **Level of expertise in qualitative research** | **Gender** |
| --- | --- | --- | --- |
| DE | GP in training and PhD-candidate (main researcher) | experienced | Female |
| MP | GP and senior researcher | expert | Female |
| YE | Professor in Meaningful Healthcare | expert | Female |
| EK | Medical oncologist, palliative care consultant, and researcher | experienced | Female |
| HS | GP and professor in General Practice | experienced | Male |
| KV | Anesthesiologist and professor in Pain and Palliative Medicine | experienced | Male |
