# Supplementary material for: Communication patterns in decision-making consultations between patients with advanced cancer and medical oncologists: A qualitative observational study
Source: PLoS One. 2026 Apr 7;21(4):e0346036. doi: 10.1371/journal.pone.0346036 (PMC13056162; doi:10.1371/journal.pone.0346036)
Supplement: S2 Table — (DOCX) [file pone.0346036.s002.docx]

**Supplementary 2 OPTION-12 items(26)**

| 1. The clinician draws attention to an identified problem as one that requires a decision-making process. 2. The clinician states that there is more than one way to deal with the identified problem (“equipoise”). 3. The clinician assesses the patient’s preferred approach to receiving information to assist decision making. 4. The clinician lists “options”, which can include the choice of ‘no action’. 5. The clinician explains the pros and cons of options to the patient (taking ‘no action’ is an option). 6. The clinician explores the patient’s expectations (or ideas) about how the problem(s) are to be managed. 7. The clinician explores the patient’s concerns (fears) about how problem(s) are to be managed. 8. The clinician checks that the patient has understood the information. 9. The clinician offers the patient explicit opportunities to ask questions during the decision-making process. 10. The clinician elicits the patient’s preferred level of involvement in decision making. 11. The clinician indicates the need for a decision-making (or deferring) stage. 12. The clinician indicates the need to review the decision (or deferment). |
| --- |
