# Supplementary material for: Communication patterns in decision-making consultations between patients with advanced cancer and medical oncologists: A qualitative observational study
Source: PLoS One. 2026 Apr 7;21(4):e0346036. doi: 10.1371/journal.pone.0346036 (PMC13056162; doi:10.1371/journal.pone.0346036)
Supplement: S3 Table — (DOCX) [file pone.0346036.s003.docx]

|  | | **Consultation number^2^** | | | | | | | | | | | | | | | |
| --- | --- | --- | --- | --- | --- | --- | --- | --- | --- | --- | --- | --- | --- | --- | --- | --- | --- |
| Theme | Category | 1 | 2 | 3 | 4 | 5 | 6 | 7 | 8 | 9 | 10 | 11 | 12 | 13 | 14 | 15 | 16 |
| 1. The medical oncologist is balancing between hope and realism | - 1. The medical oncologist presents bad news positively |  |  |  |  |  |  |  |  |  |  |  |  |  |  |  |  |
|  | - 1. The medical oncologist provides the option of anticancer treatment (even if it contradicts own insights) |  |  |  |  |  |  |  |  |  |  |  |  |  |  |  |  |
|  | - 1. The medical oncologist responds positively to the patient’s negative emotion |  |  |  |  |  |  |  |  |  |  |  |  |  |  |  |  |
|  | - 1. The medical oncologist and patient jointly navigate expectations |  |  |  |  |  |  |  |  |  |  |  |  |  |  |  |  |
| 2. There is little room for bad news | - 1. After discussing bad news, the medical oncologist and patient abruptly change the subject |  |  |  |  |  |  |  |  |  |  |  |  |  |  |  |  |
|  | - 1. The medical oncologist does not acknowledge the patient’s negative emotions |  |  |  |  |  |  |  |  |  |  |  |  |  |  |  |  |
|  | - 1. The language used by the medical oncologist masks bad news |  |  |  |  |  |  |  |  |  |  |  |  |  |  |  |  |
|  | - 1. The patient doesn’t understand the medical oncologist's message due to indirect communication |  |  |  |  |  |  |  |  |  |  |  |  |  |  |  |  |
| 3. The medical oncologist's medical perspective is leading in medical decision-making | - 1. The medical oncologist’s and patient’s perspectives differ: medical vs non-medical |  |  |  |  |  |  |  |  |  |  |  |  |  |  |  |  |
|  | - 1. The medical oncologist and patient do not discuss the patient's context in relation to decision-making |  |  |  |  |  |  |  |  |  |  |  |  |  |  |  |  |
|  | - 1. The medical oncologist leads decision-making |  |  |  |  |  |  |  |  |  |  |  |  |  |  |  |  |
|  | - 1. The patient is willing to be involved in the decision-making |  |  |  |  |  |  |  |  |  |  |  |  |  |  |  |  |
| 4. The patient and medical oncologist have a shared focus on anticancer treatment | - 1. The medical oncologist and patient reinforce each other's focus on anticancer treatment |  |  |  |  |  |  |  |  |  |  |  |  |  |  |  |  |
|  | - 1. The medical oncologist and patient attest to the positive effect of anticancer treatment on disease progression |  |  |  |  |  |  |  |  |  |  |  |  |  |  |  |  |
|  | - 1. The medical oncologist and patient or their close ones try to convince each other of the value of undergoing/continuing treatment in case of medical oncologist’s doubts |  |  |  |  |  |  |  |  |  |  |  |  |  |  |  |  |
| ^1^ Themes and categories correspond to the codebook (see Supplementary 6).  ^2^ We audio-recorded 16 consultations involving 16 patients, 8 medical oncologists and 2 medical oncology fellows.  Filled cells indicate presence across consultations, while open cells indicate absence. | | | | | | | | | | | | | | | | | |

**Supplementary 3 Theme and category^1^ presence across consultations**
