# Supplementary material for: Communication patterns in decision-making consultations between patients with advanced cancer and medical oncologists: A qualitative observational study
Source: PLoS One. 2026 Apr 7;21(4):e0346036. doi: 10.1371/journal.pone.0346036 (PMC13056162; doi:10.1371/journal.pone.0346036)
Supplement: S4 Table — (DOCX) [file pone.0346036.s004.docx]

**Supplementary 4 Patient characteristics**

|  | **Patients (N=16)** |
| --- | --- |
| Gender, *female*; N | 9 |
| Age, in years; median (min-max) | 66 (32-81) |
| Educational level;^1^ N  *Low*  *Moderate*  *High*  *Unknown* | 2  10  2  2 |
| Marital status;  *In a relationship*  *Single*  *Unknown* | 12  3  1 |
| Primary cancer diagnoses;  *Skin*  *Female genital organs*  *Urinary tract*  *Breast*  *Male genital organs*  *Bone and soft tissue*  *Head and neck*  *Endocrine glands*  *More than one cancer* | 4  3  3  1  1  1  1  1  1 |

^1^ Education is categorized as(30): high = university or higher professional education; moderate = secondary education, and; low = primary education or no education
