# Supplementary material for: Communication patterns in decision-making consultations between patients with advanced cancer and medical oncologists: A qualitative observational study
Source: PLoS One. 2026 Apr 7;21(4):e0346036. doi: 10.1371/journal.pone.0346036 (PMC13056162; doi:10.1371/journal.pone.0346036)
Supplement: S5 Table — (DOCX) [file pone.0346036.s005.docx]

**Supplementary 5 Overview of consultation content**

| **Consultation number, cancer type, gender, age^1^** | **Scan Result** | **Treatment decision** | **Summary** |
| --- | --- | --- | --- |
| 1. Breast cancer, female, 60-70 | Disease status is stable. | Continue current treatment. | The consultation began with positive news: there were no new lesions, some were stable in size, and some had reduced in size. The patient felt relieved, especially after the recent death of a neighbor had confronted her with her own mortality. The patient's relative had two questions: 1) How long can Xgeva (denosumab) be continued? 2) Should a bone scan have been performed? The medical oncologist addressed the duration of Xgeva treatment and explained the appropriate timing for a bone scan. The patient then requested prescriptions, including letrozole (hormone therapy). A follow-up appointment was scheduled, with a scan to be conducted beforehand. This suggestion came from the medical oncologist, and the patient agreed. The scheduling of the appointment took the patient's context into consideration. |
| 2. Ovarian cancer, female, 50-60 | Disease status is stable. | Continue current treatment. | The consultation began with the scan results: "Looks good." Then, physical symptoms and side effects were discussed, along with sleep and work. The medical oncologist then decided to continue the current anticancer treatment, which was discussed during the physical examination. Lastly, the medical oncologist determined the timing for the follow-up appointment. |
| 3. Ovarian cancer, female, 80-90 | Disease status is stable. | Continue current treatment. | The scan results were not discussed immediately. First, the medical oncologist and patient talked about the physical symptoms and side effect of cancer treatment. Then, the medical oncologist indicated that the result was good. The patient and medical oncologist had different interpretations of ‘good news’; the patient thought it meant the cancer was in remission, while the oncologist meant the disease was stable. The patient asked if a particular cancer treatment was an option, which it was not. The medical oncologist mentioned that several treatment options could be considered in the future. Abruptly, the consultation proceeded with a physical examination, during which small talk occurred. Finally, the medical oncologist suggested continuing the current treatment, and the patient agreed. A follow-up appointment was scheduled. |
| 4. Melanoma, male, 60-70 | Disease is progressing. | Discontinue current treatment. The decision regarding a new anticancer treatment was deferred pending a multidisciplinary meeting. | The consultation began with a discussion of the scan results; one lymph node had grown significantly, while the other lesions remained unchanged.  The medical oncologist noted that a multidisciplinary meeting would be held, and a treatment decision could only be made after this meeting. The possible outcomes discussed included radiotherapy or immunotherapy, with specific mention of which immunotherapy option might be considered. Surgery was not regarded as a viable option, though it would be addressed in the multidisciplinary meeting. The consultation then shifted to a discussion of the patient’s physical symptoms. The medical oncologist then recommended canceling the current appointment for immunotherapy administration. A follow-up appointment was scheduled. |
| 5. Ovarian cancer, female, 60-70 | Echography suggests the disease is progressive; however, further diagnostics are required to conform this. | The decision on a new anticancer treatment was postponed until after further diagnostics. | The results were not discussed immediately; the medical oncologist explained how the diagnostics were conducted. Then, bad news was presented; pathological lymph nodes were observed with a strong suspicion of cancer recurrence, but this could only be confirmed through additional diagnostics, such as a biopsy. The medical oncologist expressed a preference for a biopsy and suggested this as the next step. The medical oncologist also proposed performing a scan to assess the extent of the disease. The patient then asked questions about the diagnostic process. During this explanation, the medical oncologist outlined possible subsequent steps after diagnostics, such as chemotherapy, hormone therapy, or no anticancer treatment (e.g., supportive care). The medical oncologist advised the patient to consider these options. The patient then asked further questions about the biopsy procedure. The medical oncologist answered these questions and requested consent to proceed with additional diagnostics, which the patient grants. The consultation then moved to a physical examination, during which small talk occurred. After the physical examination, the patient inquired whether chemotherapy and immunotherapy might be options. The medical oncologist indicated that chemotherapy was a possible option and explained why immunotherapy is not. The patient then asked another question about the biopsy. The medical oncologist answered, and the patient and medical oncologist agreed on the next steps, which would be scheduled by the secretary. |
| 6. Salivary gland cancer, female, 80-90 | Disease is progressing. | Current treatment is discontinued, and a new anticancer treatment is initiated. | The consultation began with a discussion of the scan results, which showed that the patient’s symptoms were attributed to disease progressions (increased size of lymph nodes). The medical oncologist addressed pain management with painkillers. The medical oncologist then suggested radiotherapy, which would be further discussed at a multidisciplinary meeting. Additionally, the medical oncologist proposed discontinuing hormone therapy. The medical oncologist and patient briefly discussed end-of-life wishes. The medical oncologist outlined the follow-up plan: initiating radiotherapy and scheduling the next appointment in 3-4 months. |
| 7. Sarcoma, male, 60-70 | Disease is progressing. | Anticancer treatment is initiated. | The consultation began with the results: “no good news” as existing lesions in the pelvis and lung had grown, and new lesions had appeared in the lung. The medical oncologist explained that surgery was not an option (which the patient had previously inquired about). The medical oncologist proposed radiotherapy. The discussion then turned to the patient’s current condition. A plan was made for pain management (with the medical oncologist suggesting treatment options), and a follow-up was scheduled regarding radiotherapy. The medical oncologist noted that subsequent treatments could be discussed afterward. |
| 8. Merkel cell carcinoma, male, 50-60 | Disease status is stable. | Continue the current treatment strategy. | The consultation began with the results: the scan looks ‘picobello’ (perfect). The medical oncologist and patient then discussed the patient’s vacation plans. They scheduled a follow-up appointment. |
| 9. Urothelial carcinoma, female, 70-80 | Disease is progressing. | The decision is postponed until after the multidisciplinary meeting and consultation with the GP. | It was an intake. The medical oncologist began the consultation by explaining the reason for the referral. Additionally, the medical oncologist provided space for emotions. The medical oncologist then outlines the agenda. First, the medical history was reviewed. Next, physical symptoms and medication were discussed. The medical oncologist then introduced immunotherapy: its purpose, how it differed from chemotherapy, and the time required to see effects. The medical oncologist cautiously suggested that immunotherapy might no longer be an option for the patient. This led to a debate between the medical oncologist and the patient’s family. The family believed that immunotherapy should be given a chance, while the medical oncologist provided arguments to explain why it might be unwise. Ultimately, the medical oncologist proposed to measure length, weight, and blood values, consult with the GP, discuss the case in the multidisciplinary meeting, and call the patient later in the week to convey the outcome (whether or not to start immunotherapy). |
| 10. Thyroid cancer, female, 50-60 | Disease is in remission. | Continue current treatment. | The consultation began with the results: “looked very good” (lesions had decreased in size. The medical oncologist, patient, and close ones reviewed the scan. They then discussed physical symptoms, including those related to non-cancer treatments. Afterward, they talked about renewing prescriptions for non-cancer treatments. The discussion then moved to possible physical side effect of the current anticancer treatment. The medical oncologist decided to continue the treatment and scheduled a follow-up appointment, considering the patient’s context (work and vacation plans). |
| 11. Urothelial carcinoma, male, 60-70 | Disease status is stable. | Implement a treatment break from anticancer treatment. | The consultation began with the results: “It looks calm.” The patient asked for clarification on what the medical oncologist meant; all lesions were unchanged. The medical oncologist then stated that there would be a treatment break. The medical oncologist outlined what this process would involve: a scan every three months and interim follow-up consultations. The medical oncologist and patient then talked about a recently deceased loved one, how the patient was coping, and the patient’s overall well-being, including their hobbies. |
| 12. Renal cell carcinoma, male, 60-70 | Disease status is nearly stable; there is uncertainty regarding a possible new lesion, while the rest are stable or smaller. | Continue current treatment. | The results were not discussed immediately; the medical oncologist first asked how the patient was doing and then reviewed the scan in detail. The discussion of the scan was interspersed with questions about symptoms, so the results were not immediately clear. The medical oncologist then proposed not to irradiate the new area and to continue the current treatment (targeted therapy), scheduling a follow-up appointment in 3 months. The medical oncologist and patient then discussed physical symptoms, blood test results, and medication, and the medical oncologist showed the scan to the patient. |
| 13. Melanoma, male, 30-40 | Disease is in remission. | Continue current treatment. | The results were not discussed immediately; the medical oncologist first asked how the patient was doing. The medical oncologist then provided the results; the lesions had decreased in size, with some no longer visible. The medical oncologist, patient, and close ones then discussed various psychosocial and existential aspects. The patient asked about the next steps: continuing immunotherapy and a follow-up appointment in 3 months. The medical oncologist brought the consultation to a close. |
| 14. Melanoma, female, 70-80 | Disease status is stable. | Continue the current treatment strategy. | The consultation began with the results: “The scan is good” (lesions unchanged). The medical oncologist then explained what was visible on the scan. The medical oncologist outlined the next step: A PET scan in 6 months. The medical oncologist, patient, and close ones briefly discussed the anxiety the patient was experiencing regarding the potential progression of the disease. |
| 15. Renal cell carcinoma and multiple myeloma, female, 60-70 | Uncertainty about whether the disease is stable or progressing; some lesions show activity or an increase, while others do not. | The decision is pending further consultation with a colleague; however, in principle, the current strategy will be continued. | The results were not discussed immediately; the medical oncologist first asked how the patient was doing and discussed some of the patient’s physical symptoms. The results were then reviewed, which were ambiguous: some lesions showed activity or an increase, while others did not. There was uncertainty whether the disease was stable or progressing. The medical oncologist proposed radiotherapy (but mentioned they would consult with a colleague about this). The medical oncologist indicated that the current treatment would be continued for now. The medical oncologist then asked about additional symptoms and discussed the patient’s quality of life. Finally, the medical oncologist, patient, and close ones briefly talked about end-of-life wishes and the extent to which the GP was informed about these wishes. |
| 16. Prostate cancer, male, 60-70 | Disease is in remission. | Continue current treatment. | The consultation began with the results: “Good news”; all lesions had decreased. The scan was reviewed. The medical oncologist then noted indications of hydronephrosis. The medical oncologist explained that this could lead to problems in the future and discussed potential treatment options. The patient was not very concerned about this. The patient asked about an anticancer treatment option they had seen in the media. The medical oncologist indicated that this could be one of many potential options in the future. The medical oncologist and patient scheduled a follow-up appointment. Continuing the current anticancer treatment was not discussed, except that the medical oncologist did not need to provide a new prescription since the patient had enough of the medication. |
| ^1^ To maintain confidentiality, the exact ages of the patients are not disclosed. | | | |
