# Supplementary material for: Communication patterns in decision-making consultations between patients with advanced cancer and medical oncologists: A qualitative observational study
Source: PLoS One. 2026 Apr 7;21(4):e0346036. doi: 10.1371/journal.pone.0346036 (PMC13056162; doi:10.1371/journal.pone.0346036)
Supplement: S6 Table — (DOCX) [file pone.0346036.s006.docx]

**Supplementary 6 Codebook**

| Themes | Categories and axial codes^1^ |
| --- | --- |
| 1. The medical oncologist is balancing between hope and realism | - 1. The medical oncologist presents bad news positively - After delivering bad news, the medical oncologist emphasizes the positive effect of anticancer treatment - After delivering bad news, the medical oncologist indicates that anticancer treatment options are still available - Before delivering bad news, the medical oncologist expresses hope - The medical oncologist first shares the positive scan results before any negative findings   1. The medical oncologist provides the option of anticancer treatment (even if it contradicts own insights) - In the case of a patient’s/close ones’ strong desire for treatment, the medical oncologist seeks input from colleagues - In the case of a patient’s/close ones’ strong desire for treatment, the medical oncologist follows their wishes   1. The medical oncologist responds positively to the patient’s negative emotion - After the patient expresses concerns/disappointment, the medical oncologist emphasizes positive progress on the disease due to the anticancer treatment - After the patient expresses concerns, the medical oncologist indicates that anticancer treatment options are still available   1. The medical oncologist and patient jointly navigate expectations - The medical oncologist manages expectations when the patient expresses positivity regarding prognosis/anticancer treatment - The patient emphasizes positive experiences with the treatment even after the medical oncologist has tempered expectations regarding anticancer treatment |
| 2. There is little room for bad news | - 1. After discussing bad news, the medical oncologist and patient abruptly change the subject - The medical oncologist abruptly changes the subject after delivering bad news - The patient abruptly changes to a positive topic after discussing death   1. The medical oncologist does not acknowledge the patient’s negative emotions - When expressing concerns, the medical oncologist interrupts the patient - The medical oncologist rephrases the patient's statement regarding their emotional state   1. The language used by the medical oncologist masks bad news - The medical oncologist softens their use of language especially when delivering bad news and discussing end-of-life issues - The medical oncologist uses many words when explaining bad news   1. The patient doesn’t understand the medical oncologist's message due to indirect communication - The medical oncologist does not directly disclose the scan result - After concluding with a summary, the medical oncologist provides the information needed for shared decision-making - The medical oncologist uses technical language when explaining the results of diagnostic tests - The medical oncologist contradicts previously given information - The medical oncologist explains something while indicating that it is not actually relevant - The patient draws an own conclusion that there is bad news |
| 3. The medical oncologist's medical perspective is leading in medical decision-making | - 1. The medical oncologist’s and patient’s perspectives differ: medical vs non-medical - The medical oncologist responds to the patient's psycho-emotional/existential question/comment from a medical perspective - The medical oncologist doesn’t address the patient's or close ones’ comments about what is important to them in decision-making - The patient or their close ones reiterate what concerns them - The patient interprets "good" news differently from the medical oncologist   1. The medical oncologist and patient do not discuss the patient's context in relation to decision-making - The medical oncologist and patient discuss the patient's context - The medical oncologist considers the patient's context when planning follow-up appointments   1. The medical oncologist leads decision-making - The medical oncologist decides - The medical oncologist proposes a treatment plan - The medical oncologist asks for approval for the proposed treatment plan - The patient agrees with the medical oncologist's proposed treatment plan - The patient or their close ones are satisfied when the medical oncologist is   1. The patient is willing to be involved in the decision-making - The patient asks for clarification when something hasn’t been clearly explained - The patient or their close ones ask for the information needed for decision-making - The patient or their close ones weigh the pros and cons of the treatment - The patient indicates that the medical oncologist’s proposed treatment plan aligns with their own expectations - The patient or close one adopts the medical oncologist's language |
| 4. The patient and medical oncologist have a shared focus on anticancer treatment | - 1. The medical oncologist and patient reinforce each other's focus on anticancer treatment - The patient or their close ones feel they have to take action regarding their treatment - The patient feels they have no choice - The medical oncologist confirms the patient’s lack of choice - The medical oncologist doesn’t respond to the patient’s referral to ‘having no choice’ - The medical oncologist indicates that the patient always has a choice, but ... - The medical oncologist asks if the anticancer treatment has had a positive effect   1. The medical oncologist and patient attest to the positive effect of anticancer treatment on disease progression - The medical oncologist highlights the (unexpected) positive effects of anticancer treatment on disease progression - The patient or their close ones confirm a positive effect of anticancer treatment on disease progression   1. The medical oncologist and patient or their close ones try to convince each other of the value of undergoing/continuing treatment in case of medical oncologist’s doubts - The medical oncologist attempts to make the patient or their close ones realize the poor prognosis (when the patient/close one strongly desires anticancer treatment) - In response to the medical oncologist stating that a specific anticancer treatment has no medical benefit, the close ones review an earlier statement about the patient’s condition |
| ^1^ Open coding results are on request available. Not all axial codes are elaborated in the results section of the present study | |
