# Supplementary material for: Communication patterns in decision-making consultations between patients with advanced cancer and medical oncologists: A qualitative observational study
Source: PLoS One. 2026 Apr 7;21(4):e0346036. doi: 10.1371/journal.pone.0346036 (PMC13056162; doi:10.1371/journal.pone.0346036)
Supplement: Attachment — Submitted filename: Response to Reviewers.docx [file pone.0346036.s008.docx]

We want to thank the reviewers and editor for reviewing our paper. We highly appreciate the constructive feedback that encouraged us to improve the manuscript. Below, we describe our reaction to the comments.

# Reviewer 1:

***1. The applied Reflexive Thematic Analysis (of what consists this method) and the way the researchers proceeded with this method is not or poorly described.***

*Reaction:* We have clarified both the methodological approach and our reflexive stance in the Methods section. While the study was originally reported using COREQ, we also considered the Reflexive Thematic Analysis Reporting Guidelines (RTARG, Braun & Clarke 2024, Palliat Med.) to enhance clarity and completeness. Accordingly, we have now clarified our rationale for using RTA and more comprehensively described our reflexivity.

Methods, Study Design, page 5, lines 76-86

In this qualitative observational study, we applied reflexive thematic analysis to audio-recorded outpatient consultations of the Medical Oncology department. **Reflexive thematic analysis was chosen because it allows for an in-depth exploration of communication processes and the identification of recurring patterns, while acknowledging the active role of the researchers in interpreting meaning. This approach was particularly suited to our aim of understanding how shared decision-making unfolds in consultations with patients with advanced cancer.** We used the Consolidated Criteria for Reporting Qualitative Research (COREQ) to guide reporting of this study.(25) **In addition, we considered the Reflexive Thematic Analysis Reporting Guidelines (RTARG) to enhance clarity and completeness.(26)** The research ethics committee of Radboud University Medical Center judged the study to be fully compliant with the Dutch Medical Research Involving Human Subjects Act (case number 2018-4992).

Methods, Study Team and Reflexivity, page 6, lines 106-114

Team members had diverse research and clinical backgrounds, including primary care, palliative care, and medical oncology (investigator triangulation). The team also included a professor in Meaningful Healthcare with expertise in contextual communication and care. Additionally, all team members had prior experience conducting qualitative research in clinical settings. Further information on the study team and reflexivity is provided in Supplementary 1.

**We acknowledge that our perspectives influenced the attention to communication patterns. For example, DE had substantial prior experience observing oncology consultations(28) and conducting a qualitative embedded multiple-case study on shared decision-making(24), which guided the reflexive interpretation of the data. Moreover, the team was largely composed of general practitioners and palliative care physicians, for whom communication is a core element of their clinical practice. To account for these influences, we engaged in reflexive practices throughout the analysis, including notetaking during coding, iterative code review, and multiple team discussions. These strategies helped ensure that the resulting themes were grounded in the data rather than solely shaped by researchers’ preconceptions.**

Methods, Data Analysis, pages 7-8, lines 151-155

Transcripts were imported into ATLAS.ti (version 23) for reflexive thematic analysis.(34) DE read all transcripts and inductively coded concepts as closely as possible to the participant’s words to minimize subjectivity. Between October 2023 and February 2024, DE and MP iteratively discussed the codes during weekly meetings until consensus was reached. If new codes emerged, all consultations were reviewed in relation to the latest codes. Next, DE and MP grouped similar concepts into initial categories and themes, resulting in a preliminary codebook. **During the first research meeting, the authors reviewed the codes to ensure they reflected the data closely, with minimal interpretation or judgment. In the second meeting, the authors similarly ensured that the creation of categories and themes did not introduce excessive interpretation, and consensus on the final codebook was reached with the full team.** Investigator triangulation helped to ensure that the themes reflected the full range and depth of the data.(35) Additionally, we evaluated the consistency of constructed themes across consultations (Supplementary 3) and found that each theme was widely supported, thereby validating the themes created.

***2. Background of the researchers: "meaningful communication"? This is not a profession like the professions of the other researchers (palliative care, medical oncology, primary care), what is the professional background?***

*Reaction:* To clarify, the research team included a professor in Meaningful Healthcare with expertise in contextual communication and care, alongside members with clinical backgrounds in primary care, palliative care, and medical oncology. We have revised the text in the Methods section accordingly to avoid ambiguity.

Methods, Study Team and Reflexivity, page 6, lines 102-103

Team members had diverse research and clinical backgrounds, including primary care, palliative care, and medical oncology (investigator triangulation). **The team also included a professor in Meaningful Healthcare with expertise in contextual communication and care.** Additionally, all team members had prior experience conducting qualitative research in clinical settings. Further information on the study team and reflexivity is provided in Supplementary 1.

***3. Reading the discussion it clearly lacks expertise in and input from liaison psychiatry/psycho-oncology: why did the researcher not associate a professional with this background?***

*Reaction:* We agree that expertise in (medical) psychology and behavioral science could have provided additional perspectives, not only on the psychological dimensions of shared decision-making but also on the design and interpretation of medical education interventions, as now discussed in the manuscript. Our study team was largely composed of general practitioners and palliative care physicians, for whom communication is a core clinical and professional competence. While we have knowledge of psychological and behavioral principles, we are not experts in psychology or behavioral science. During the analysis phase, we considered involving such expertise; however, this was not feasible within our study team, particularly given the exploratory nature of the study. To acknowledge this limitation, we have now added a statement in the limitations section and recommend that future research incorporate perspectives from medical) psychology and behavioral science to further enrich the understanding and interpretation of communication processes in oncology consultations.

Discussion, pages 18-19, lines 280-288

Several limitations should be considered. First, shared decision-making is dynamic and continuous, yet our observations were limited to consultations in which patients received scan results. Consequently, we could not account for earlier discussions between medical oncologists and patients that may have influenced these decisions. Nonetheless, consultations where patients receive scan results are pivotal in initiating the decision-making process regarding whether to continue, discontinue or start treatment. **Second, our research team did not include expertise in (medical) psychology or behavioral science, disciplines that have strongly informed medical communication education and could have provided additional perspectives on the psychological and interactional mechanisms underlying the created themes and self-reinforcing cycle. Furthermore, medical education expertise, particularly in medical communication, could have enriched our analysis by illuminating how communication skills are taught, learned, and enacted in practice. We therefore recommend that future research integrate these disciplines alongside clinical and communication-focused expertise, enabling a more comprehensive understanding of the process shaping shared decision-making in oncology consultations.**

***4. Why do the authors describe the questionnaires? This is a qualitative study, and there is no articulation between the quantitative and qualitative data?***

*Reaction:* We included the OPTION-12 scores and post-consultation questionnaires to provide contextual information about the extent of shared decision-making and satisfaction from both patients and oncologists. While our primary analysis is qualitative, these quantitative measures help interpret the communication patterns we observed by showing how the participants experienced the consultations. The quantitative data were analysed descriptively and are presented alongside the qualitative findings to enrich the interpretation, rather than to perform a formal mixed-methods comparison. We have now clarified this purpose and integration in the manuscript.

Methods, Data Collection Consultation Context Measures and Participant Characteristics, pages 6-7, lines 124-125

**To explore the heterogeneity of our convenience sample, we collected quantitative data describing the participants and the consultations.** Consultations were assessed in terms of the extent of shared decision-making and participants’ immediate perceptions. Each consultation was scored by the first author (DE) using the OPTION-12 instrument (Supplementary 2)(29). Immediately following the consultation, patients and medical oncologists (fellows) completed a brief questionnaire assessing their satisfaction with the consultation, including the extent of shared decision-making. Basic characteristics of both patients and medical oncologists were also collected.

Patient Questionnaires included:

- SDM-Q-9 (30, 31) to assess the patient’s involvement in decision-making.
- VAS (Visual Analogue Scales)(32) to rate satisfaction with shared decision-making and general satisfaction with the consultation.

Medical Oncologist Questionnaires included:

- VAS scores for satisfaction with shared decision-making and general satisfaction with the consultation.
- Estimates of patient satisfaction with decision-making and general satisfaction with the consultation.

Participant Characteristics documented were:

- Patients: gender, age, type of cancer, educational level (33), and marital status.
- Medical oncologists: gender and work experience.

***5. The discussion seems to me rather superficial and this is a shame, since the study is very interesting; to give a few examples: a) SDM is not the only objective of a consultation, crtitique towards the oncologists have therefore be attenuated, and SDM should be placed within the context of all other objectives (e.g., support); b) some phenomena such as the shared focus on anticancer treatment can be explained by collusion (see the recent reviews and studies by Stiefel et al.), and if collusion is at work, simply feedback the observation to oncologists will not do it (see the beforementioned (for approaches, see these papers); c) other reactions of oncologists such as presenting bad news positively or responding to patient's negative emotions positively is understandable when one turns to the psychology of the clinician (e.g., feelings of guilt to harm the patient psychologically, fear of the patient's emotions - see for example the recent ESMO Guidelines on communication in chronic cancer care, in which an important part is dedicated to the "psychology of the clinician"; d) the interconnection of the four findings should also be discussed in light of the psychology of the patient, of the clinician and their interactional dynamics. a) - d) is important to discuss, since the suggested ways to improve the situation will depend on. Again, just to restore these finding to oncologists will not modify their communication behaviour, since it's not about technique but psychology; psycho-oncology has means to modify these behaviours (communication training, individual and group supervision, etc.***

*Reaction*: We agree that shared decision-making is not the only objective of oncology consultations, and that psychological and interactional factors likely contribute to the communication patterns we observed.

1. Multiple consultation objectives: Our quantitative results showed a discrepancy between objectively measured shared decision-making (OPTION-12 scores) and patient- and physician-reported experiences, with the latter generally high despite relatively low OPTION-12 scores. This pattern, also reported by Geessink et al., Kasper et al., and Scholl et al.*, suggests that support, empathy, and other relational aspects play an important role alongside shared decision-making. However, in our study, we used these quantitative measures primarily to provide context for the consultations and to explore heterogeneity across cases. Importantly, the quantitative data do not address our primary study aim, which was to identify communication patterns in decision-making conversations between patients with advanced cancer and medical oncologists to enable more person-centered palliative care through improved shared decision-making. For this reason, we did not include these quantitative results in the Discussion, as we believe doing so would distract from the qualitative insights central to our research question.

* Geessink NH, Ofstad EH, Olde Rikkert MGM, van Goor H, Kasper J, Schoon Y. Shared decision-making in older patients with colorectal or pancreatic cancer: Determinants of patients' and observers' perceptions. Patient Educ Couns. 2018;101(10):1767-74.

Kasper J, Hoffmann F, Heesen C, Kopke S, Geiger F. Completing the third person's perspective on patients' involvement in medical decision-making: approaching the full picture. Z Evid Fortbild Qual Gesundhwes. 2012;106(4):275-83.

Scholl I, Kriston L, Dirmaier J, Harter M. Comparing the nine-item Shared Decision-Making Questionnaire to the OPTION Scale - an attempt to establish convergent validity. Health Expect. 2015;18(1):137-50.

2. Psychological explanations: The observed phenomena, such as the shared focus on anticancer treatment, can plausibly be explained through psychological perspectives, including collusion, clinician emotions (e.g., balancing hope and realism, fear of causing distress), and interactional dynamics. We have now incorporated this discussion in the revised manuscript.
3. Future research and interventions: As our study team did not include psychologists or behavioral scientists, we recommend that future research integrate these perspectives. For example, multidisciplinary reflection on video-recorded consultations could help interpret themes in light of patient and clinician psychology, while an educational perspective could inform the design of effective training strategies. Interventions such as targeted supervision, communication training, or structured approaches to collusion could then be evaluated and embedded in medical education and continuing professional development.

These revisions strengthen the discussion by situating our findings within broader psychological and relational contexts, clarifying the interconnections of the observed themes, and suggesting avenues for more effective intervention.

Discussion, pages 18-20, lines 269-273 and 307-311

(…)

**Several psychological and interactional processes likely underpin the four themes and the self-reinforcing cycle, including clinicians’ psychology, patients’ psychology, and their interactional dynamics—for example, collusions between patients and clinicians.(37, 38) Understanding how these processes interact, as emphasized in, e.g., (medical) psychology and behavioral science, is crucial and warrants further study, with our observations providing an important initial step toward this goal.**

(…)

Follow-up to our study offers a valuable opportunity to integrate research with clinical practice and education. Audio- or video-recorded consultations can be reviewed to provide feedback to healthcare professionals, either collaboratively with patients, as demonstrated in studies on medically unexplained symptoms (41), or internally during team review sessions. Previous research shows that patient-collected recordings with structured feedback can enhance physicians' attention to contextual factors, improve outcomes, reduce hospitalizations, and lower healthcare costs.(42) **Beyond feedback, supervision offers a promising avenue for addressing the psychological and interactional dynamics, including mechanisms such as collusion and the psychology of patients and clinicians.(37, 38) Further research is needed to determine whether these approaches can effectively disrupt the cycle and strengthen shared decision-making within palliative care.** Ultimately, such insights could inform clinical guidelines and medical education, better preparing healthcare professionals for person-centered consultations.

# Reviewer 2:

***6. Introduction: Well-written overall. "Vicious" cycle: the how underlying this is unclear to me. Discussion of this and elaboration in the introduction would be useful. Some discussion of the mechanisms by which this might happen might also be helpful for next steps in intervention.***

*Reaction*: We have replaced “vicious cycle” with “self-reinforcing cycle,” as we believe this subtle change better captures the concept. Additionally, we have slightly revised the text under “Theme interrelations” to better explain how the shared focus on anticancer treatment relates to balancing hope and realism. Inspired by this comment and feedback from Reviewer 1, we also added a limitation noting that our study team did not include expertise in psychology or behavioral science, which could have provided relevant insights into the underlying mechanisms of the observed cycle—especially since several phenomena (as noted by Reviewer 1) can be explained by psychological and interactional processes. We hope these changes adequately address the reviewer’s concerns.

Results, page 17, lines 246-255

*Theme interrelations: a* ***self-reinforcing cycle*** *unveiled*

Our analysis and the combined quotations in Table 2 suggested an interconnection between the four themes, forming **a self-reinforcing cycle** (see Figure 1). Efforts by medical oncologists to balance hope and realism influenced how bad news was delivered, often diluting the message. This dilution could make it more challenging for patients to engage as equal partners in decision-making. Consequently, the medical oncologist’s perspective tended to dominate the conversation, likely limiting consideration of the patient’s viewpoint and reinforcing a shared focus on anticancer treatment options. When this focus became overwhelming, medical oncologists appeared to struggle to temper it, which in turn made balancing hope and realism even more challenging in subsequent interactions. This dynamic created a feedback loop, whereby each theme amplified the others, sustaining the cycle over time.

Discussion, pages 18-19, lines 280-288

Several limitations should be considered. First, shared decision-making is dynamic and continuous, yet our observations were limited to consultations in which patients received scan results. Consequently, we could not account for earlier discussions between medical oncologists and patients that may have influenced these decisions. Nonetheless, consultations where patients receive scan results are pivotal in initiating the decision-making process regarding whether to continue, discontinue or start treatment. **Second, our research team did not include expertise in (medical) psychology or behavioral science, disciplines that have strongly informed medical communication education and could have provided additional perspectives on the psychological and interactional mechanisms underlying the created themes and self-reinforcing cycle. Furthermore, medical education expertise, particularly in medical communication, could have enriched our analysis by illuminating how communication skills are taught, learned, and enacted in practice. We therefore recommend that future research integrate these disciplines alongside clinical and communication-focused expertise, enabling a more comprehensive understanding of the process shaping shared decision-making in oncology consultations.**

***7. Results & Discussion: The authors make points, such as "no room for bad news". It would be helpful to comment on literature and/or theoretical frameworks that suggest why this might be the case? Is there a wider societal context at play here? Or is it medical oncologists' own values? How might these intersect? Thinking about what these codes and findings might mean in terms of how we deliver care might deepen the insights provided by the manuscript.***

*Reaction:* We note that this aligns closely with Reviewer 1’s comment 5, which also encouraged discussion of psychological and interactional factors underlying the observed phenomena. In response, we have revised the Discussion to acknowledge these perspectives, highlighting mechanisms such as collusion and clinicians’ psychology, and we emphasize the importance of integrating expertise from psycho-oncology and behavioral science in future research. These changes aim to provide a deeper understanding of the findings and their implications for how care is delivered.

Discussion, pages 18-20, lines 269-273 and 307-311

(…)

**Several psychological and interactional processes likely underpin the four themes and the self-reinforcing cycle, including clinicians’ psychology, patients’ psychology, and their interactional dynamics—for example, collusions between patients and clinicians.(37, 38) Understanding how these processes interact, as emphasized in, e.g., (medical) psychology and behavioral science, is crucial and warrants further study, with our observations providing an important initial step toward this goal.**

(…)

Follow-up to our study offers a valuable opportunity to integrate research with clinical practice and education. Audio- or video-recorded consultations can be reviewed to provide feedback to healthcare professionals, either collaboratively with patients, as demonstrated in studies on medically unexplained symptoms (41), or internally during team review sessions. Previous research shows that patient-collected recordings with structured feedback can enhance physicians' attention to contextual factors, improve outcomes, reduce hospitalizations, and lower healthcare costs.(42) **Beyond feedback, supervision offers a promising avenue for addressing the psychological and interactional dynamics, including mechanisms such as collusion and the psychology of patients and clinicians.(37, 38) Further research is needed to determine whether these approaches can effectively disrupt the cycle and strengthen shared decision-making within palliative care.** Ultimately, such insights could inform clinical guidelines and medical education, better preparing healthcare professionals for person-centered consultations.

***8. The paper itself might benefit from consideration of theory or theoretical frameworks that elaborate on decision-making. What sort of shared decision-making models have already been suggested? How do the findings compare? Do they map onto similar domains or do they deviate significantly?***

*Reaction: In the revised Introduction, we have now incorporated existing theoretical frameworks for shared decision-making, including the model of Elwyn et al. (choice talk, option talk, and decision talk) and the refinements proposed by Stiggelbout et al. We briefly outline these models and position our study in relation to them, noting that despite the availability of such frameworks, shared decision-making remains challenging in daily oncology practice.*

Introduction, page 3, lines 47-53

(…)

**Several models of shared decision-making have been proposed to support this process. A widely used framework is that of Elwyn et al.(6), which distinguishes three key steps: *choice talk* (making clear that a decision exists), *option talk* (presenting available options and their pros and cons), and *decision talk* (supporting patients in exploring preferences and reaching a decision). Stiggelbout et al.(7) have, amongst others, further refined this model by emphasizing deliberation about patient preferences and the patient’s preferred role in decision-making. Despite the availability of such theoretical frameworks, the application of shared decision-making in daily oncology practice remains limited. (1, 8-10)**

(…)

***9. I would have liked to also understand a little more about the reflexive approach applied by the authors - what were the positions they approached the analysis with? What were their biases, strengths and limitations?***

*Reaction:* This comment relates to the same point as Reviewer 1, Comment 1, regarding the description and application of our reflexive thematic analysis. Please see our response to Reviewer 1, Comment 1, where we have clarified both the procedural steps and our reflexive stance, including researcher positioning, biases, and strategies to ensure rigor.

Methods, Study Design, page 5, lines 76-86

In this qualitative observational study, we applied reflexive thematic analysis to audio-recorded outpatient consultations of the Medical Oncology department. **Reflexive thematic analysis was chosen because it allows for an in-depth exploration of communication processes and the identification of recurring patterns, while acknowledging the active role of the researchers in interpreting meaning. This approach was particularly suited to our aim of understanding how shared decision-making unfolds in consultations with patients with advanced cancer.** We used the Consolidated Criteria for Reporting Qualitative Research (COREQ) to guide reporting of this study.(25) **In addition, we considered the Reflexive Thematic Analysis Reporting Guidelines (RTARG) to enhance clarity and completeness.(26)** The research ethics committee of Radboud University Medical Center judged the study to be fully compliant with the Dutch Medical Research Involving Human Subjects Act (case number 2018-4992).

Methods, Study Team and Reflexivity, page 6, lines 106-114

Team members had diverse research and clinical backgrounds, including primary care, palliative care, and medical oncology (investigator triangulation). The team also included a professor in Meaningful Healthcare with expertise in contextual communication and care. Additionally, all team members had prior experience conducting qualitative research in clinical settings. Further information on the study team and reflexivity is provided in Supplementary 1.

**We acknowledge that our perspectives influenced the attention to communication patterns. For example, DE had substantial prior experience observing oncology consultations(28) and conducting a qualitative embedded multiple-case study on shared decision-making(24), which guided the reflexive interpretation of the data. Moreover, the team was largely composed of general practitioners and palliative care physicians, for whom communication is a core element of their clinical practice. To account for these influences, we engaged in reflexive practices throughout the analysis, including notetaking during coding, iterative code review, and multiple team discussions. These strategies helped ensure that the resulting themes were grounded in the data rather than solely shaped by researchers’ preconceptions.**

Methods, Data Analysis, pages 7-8, lines 151-155

Transcripts were imported into ATLAS.ti (version 23) for reflexive thematic analysis.(34) DE read all transcripts and inductively coded concepts as closely as possible to the participant’s words to minimize subjectivity. Between October 2023 and February 2024, DE and MP iteratively discussed the codes during weekly meetings until consensus was reached. If new codes emerged, all consultations were reviewed in relation to the latest codes. Next, DE and MP grouped similar concepts into initial categories and themes, resulting in a preliminary codebook. **During the first research meeting, the authors reviewed the codes to ensure they reflected the data closely, with minimal interpretation or judgment. In the second meeting, the authors similarly ensured that the creation of categories and themes did not introduce excessive interpretation, and consensus on the final codebook was reached with the full team.** Investigator triangulation helped to ensure that the themes reflected the full range and depth of the data.(35) Additionally, we evaluated the consistency of constructed themes across consultations (Supplementary 3) and found that each theme was widely supported, thereby validating the themes created.

***10. Lastly quantitative measures have been included in the paper write up but I'm not sure how these compare to the qualitative data. Making this clearer and how both sources of data have been analysed would help clarify the findings presented in the research.***

*Reaction:* This comment raises the same point as Reviewer 1, Comment 4 regarding the inclusion and interpretation of quantitative measures. Please see our detailed response to Reviewer 1, Comment 4 for our explanation and corresponding revisions: We included the OPTION-12 scores and post-consultation questionnaires to provide contextual information about the extent of shared decision-making and satisfaction from both patients and oncologists. While our primary analysis is qualitative, these quantitative measures help interpret the communication patterns we observed by showing how the participants experienced the consultations. The quantitative data were analysed descriptively and are presented alongside the qualitative findings to enrich the interpretation, rather than to perform a formal mixed-methods comparison. We have now clarified this purpose and integration in the manuscript.

Methods, Data Collection Consultation Context Measures and Participant Characteristics, pages 6-7, lines 124-125

**To explore the heterogeneity of our convenience sample, we collected quantitative data describing the participants and the consultations.** Consultations were assessed in terms of the extent of shared decision-making and participants’ immediate perceptions. Each consultation was scored by the first author (DE) using the OPTION-12 instrument (Supplementary 2)(29). Immediately following the consultation, patients and medical oncologists (fellows) completed a brief questionnaire assessing their satisfaction with the consultation, including the extent of shared decision-making. Basic characteristics of both patients and medical oncologists were also collected.

Patient Questionnaires included:

- SDM-Q-9 (30, 31) to assess the patient’s involvement in decision-making.
- VAS (Visual Analogue Scales)(32) to rate satisfaction with shared decision-making and general satisfaction with the consultation.

Medical Oncologist Questionnaires included:

- VAS scores for satisfaction with shared decision-making and general satisfaction with the consultation.
- Estimates of patient satisfaction with decision-making and general satisfaction with the consultation.

Participant Characteristics documented were:

- Patients: gender, age, type of cancer, educational level (33), and marital status.
- Medical oncologists: gender and work experience.

Journal Requirements:

***11. Please ensure that your manuscript meets PLOS ONE's style requirements, including those for file naming.***

**The PLOS ONE style templates can be found at**

[**https://journals.plos.org/plosone/s/file?id=wjVg/PLOSOne_formatting_sample_main_body.pdf**](https://journals.plos.org/plosone/s/file?id=wjVg/PLOSOne_formatting_sample_main_body.pdf) **and**

[**https://journals.plos.org/plosone/s/file?id=ba62/PLOSOne_formatting_sample_title_authors_affiliations.pdf**](https://track.editorialmanager.com/CL0/https:%2F%2Fjournals.plos.org%2Fplosone%2Fs%2Ffile%3Fid=ba62%2FPLOSOne_formatting_sample_title_authors_affiliations.pdf/1/010f01985fdf6b73-6144c65a-f143-4fa9-a870-5cd60994e59a-000000/IgP8xcCNbTmqL0rFQerMTuyKY8f99f4HIvQ67gDDl1U=222)

*Reaction:* We have carefully reviewed the PLOS ONE style templates and made the necessary adjustments to ensure that the manuscripts and associated files meet the journal’s formatting requirements.

***12.*** [***Thank you for stating in your Funding Statement:
“D.J.M. Ermers was supported by a personal grant from the Radboudumc. The funders had no role in study design, conduct, analysis, or reporting.” Please provide an amended statement that declares *all* the funding or sources of support (whether external or internal to your organization) received during this study, as detailed online in our guide for authors at http://journals.plos.org/plosone/s/submit-now. Please also include the statement “There was no additional external funding received for this study.” in your updated Funding Statement.
Please include your amended Funding Statement within your cover letter. We will change the online submission form on your behalf.***](Thank%20you%20for%20stating%20in%20your%20Funding%20Statement:)

*Reaction:* We have included the following statement in our cover letter: ***“***D.J.M. Ermers was supported by a personal grant from the Radboud university medical centre (RvBI7.52147). The funders had no role in study design, data collection and analysis, decision to publish, or preparation of the manuscript. There was no additional external funding received for this study.”

***13. In the online submission form, you indicated that [The sensitivity and confidentiality of the raw qualitative data makes sharing of the data without compromising confidentiality and privacy impossible, therefore consent for sharing of the raw data was not asked from the participants. Where possible, the raw qualitative data will be anonymized by data aggregation to enable sharing for reuse. The anonymous data used for analysis are available from the corresponding author and the research unit in CC (***[***Onderzoek.anes@radboudumc.nl***](mailto:Onderzoek.anes@radboudumc.nl)***) upon reasonable request.].
All PLOS journals now require all data underlying the findings described in their manuscript to be freely available to other researchers, either 1. In a public repository, 2. Within the manuscript itself, or 3. Uploaded as supplementary information.
This policy applies to all data except where public deposition would breach compliance with the protocol approved by your research ethics board. If your data cannot be made publicly available for ethical or legal reasons (e.g., public availability would compromise patient privacy), please explain your reasons on resubmission and your exemption request will be escalated for approva****l.*

*Reaction:* To clarify, the anonymized data underlying the findings are freely available within the manuscript. However, the raw qualitative data (full transcripts) cannot be shared publicly, as doing so would compromise participant (patients’ as well as medical oncologists’) confidentiality and privacy. Consent for public sharing of raw data was therefore not obtained. Where possible, raw qualitative data will be further anonymized through data aggregation to enable reuse. The anonymized data used for analysis are available from the corresponding author and the research unit in CC (Onderzoek.anes@radboudumc.nl) upon reasonable request.

***14. Your ethics statement should only appear in the Methods section of your manuscript. If your ethics statement is written in any section besides the Methods, please delete it from any other section.***

*Reaction: As requested, we have removed the ethics statement from all sections except the Methods section.*

***15. We note that Figure 1 in your submission contain copyrighted images. All PLOS content is published under the Creative Commons Attribution License (CC BY 4.0), which means that the manuscript, images, and Supporting Information files will be freely available online, and any third party is permitted to access, download, copy, distribute, and use these materials in any way, even commercially, with proper attribution. For more information, see our copyright guidelines:*** [***http://journals.plos.org/plosone/s/licenses-and-copyright***](http://journals.plos.org/plosone/s/licenses-and-copyright)***.

We require you to either (1) present written permission from the copyright holder to publish these figures specifically under the CC BY 4.0 license, or (2) remove the figures from your submission:

a. You may seek permission from the original copyright holder of Figure 1 to publish the content specifically under the CC BY 4.0 license.

We recommend that you contact the original copyright holder with the Content Permission Form (***[***http://journals.plos.org/plosone/s/file?id=7c09/content-permission-form.pdf***](http://journals.plos.org/plosone/s/file?id=7c09/content-permission-form.pdf)***) and the following text:
“I request permission for the open-access journal PLOS ONE to publish XXX under the Creative Commons Attribution License (CCAL) CC BY 4.0 (***[***http://creativecommons.org/licenses/by/4.0/***](http://creativecommons.org/licenses/by/4.0/)***). Please be aware that this license allows unrestricted use and distribution, even commercially, by third parties. Please reply and provide explicit written permission to publish XXX under a CC BY license and complete the attached form.”

Please upload the completed Content Permission Form or other proof of granted permissions as an "Other" file with your submission.

In the figure caption of the copyrighted figure, please include the following text: “Reprinted from [ref] under a CC BY license, with permission from [name of publisher], original copyright [original copyright year].”

b. If you are unable to obtain permission from the original copyright holder to publish these figures under the CC BY 4.0 license or if the copyright holder’s requirements are incompatible with the CC BY 4.0 license, please either i) remove the figure or ii) supply a replacement figure that complies with the CC BY 4.0 license. Please check copyright information on all replacement figures and update the figure caption with source information. If applicable, please specify in the figure caption text when a figure is similar but not identical to the original image and is therefore for illustrative purposes only.***

*Reaction:* I (first author) created the original figure and adapted four licensed icons from the Noun Project to fit the illustration. The purchased licenses permit use without attribution, and proof of these licenses is attached.

***16.*** [***Please include captions for your Supporting Information files at the end of your manuscript, and update any in-text citations to match accordingly. Please see our Supporting Information guidelines for more information: http://journals.plos.org/plosone/s/supporting-information.***](Please%20include%20captions%20for%20your%20Supporting%20Information%20files%20at%20the%20end%20of%20your%20manuscript,%20and%20update%20any%20in-text%20citations%20to%20match%20accordingly.%20Please%20see%20our%20Supporting%20Information%20guidelines%20for%20more%20information:%20http:/journals.plos.org/plosone/s/supporting-information.%20)

*Reaction:* We have included captions for all Supporting Information files at the end of our manuscript and updated the in-text citations to match accordingly.

***17. If the reviewer comments include a recommendation to cite specific previously published works, please review and evaluate these publications to determine whether they are relevant and should be cited. There is no requirement to cite these works unless the editor has indicated otherwise.***

*Reaction:* We have reviewed and evaluated the recommended publications and have cited those we considered relevant to our study.
